# Positional Profiling of Anthropometric, Baropodometric, and Grip Strength Traits in Male Volleyball Players: Insights from a National Colombian Study

**DOI:** 10.3390/jfmk10020197

**Published:** 2025-05-29

**Authors:** Adrián De la Rosa, María Alejandra Camacho-Villa, Fernando Millan-Domingo, Juan Carlos Saavedra, Marina Politi Okoshi, Luana Urbano Pagan

**Affiliations:** 1 Freshage Research Group, Department of Physiology, Faculty of Medicine, University of Valencia, CIBERFES, Fundación Investigación Hospital Clínico Universitario/INCLIVA, 46010 Valencia, Spain; fernando.millan-domingo@uv.es; 2 Body, Physical Activity and Sport Study Group (GECAFD), Sports Department, Universidad Industrial de Santander, Bucaramanga 680002, Colombia; 3 Harpeer Research Group, Yumbo 760001, Colombia; alejacvilla@gmail.com; 4 Pain Study Group (GED), Physical Therapy School, Universidad Industrial de Santander, Bucaramanga 680002, Colombia; 5 GCED Research Group, Bucaramanga 688006, Colombia; juancs19@hotmail.com; 6 Botucatu Medical School, São Paulo State University, Botucatu 18618-970, Brazil; marina.okoshi@unesp.br (M.P.O.); luana.pagan@unesp.br (L.U.P.)

**Keywords:** upper limb variables, positional differences, baropodometry, volleyball performance

## Abstract

**Background:** In volleyball, upper limb dimensions, handgrip strength (HGS), and baropodometric parameters are critical for executing offensive and defensive actions during the match. These movements demand not only physical precision but also carry a significant risk of injury, varying by playing position. **Objectives:** This study aimed to determine the differences in specific upper limb anthropometric characteristics, HGS, and selected baropodometric variables among U-23 male volleyball players concerning playing position. **Methods:** The sample consisted of 92 U-23 male players who prepared for the U-23 Men’s Volleyball National Championship 2022 (20.39 (1.74) years, 184 (8.46) cm, 75.52 (10.20) kg). Playing positions analyzed were setters (*n* = 12), outside (*n* = 18), opposites (*n* = 19)*,* middle blockers (*n* = 16), and liberos *(n* = 12). **Results:** player position differences in HGS and several anthropometric upper limb variables were observed. Middle blockers, outsides, and opposites exhibited superior anthropometric traits in most of the measurements compared to liberos and setters (*p* < 0.05). Differences in baropodometric parameters were only found between feet and their zones when the entire sample was evaluated. Finally, regression analysis identified dominant hand breadth (β = 3.42, 95%CI [0.43, 6.40], upper arm muscle area (β = 0.157, 95%CI [0.02, 0.29]), and wrist diameter (β = 3.59, IC 95% [0.49, 6.68]) as associated variables of HGS. **Conclusions:** The study underscores the importance of positional profiling in volleyball, revealing key physical traits linked to performance. The observed differences are likely attributable to the specific role and physical demands inherent to each playing position. These findings can guide targeted training and injury prevention strategies to enhance performance.

## 1. Introduction

Volleyball players are expected to exhibit specific functional and structural characteristics that significantly influence their athletic success and performance [1,2,3]. Several anthropometric measurements, including height, body composition, and segmental length of body parts, particularly the upper limbs, play a crucial role in skill execution and team strategy [3,4].

In volleyball, as in other ball sports (i.e., basketball, softball, and handball) where hand–ball interaction is fundamental, numerous movements rely on the continuous use of wrist and digit flexors, particularly in attacking, blocking, and serving [5,6]. HGS emerges as another physical factor influencing these specific movements, serving as a key determinant of optimal performance and providing athletes with considerable advantages during gameplay [7,8,9].

Previous studies have examined [1,10,11,12] the anthropometric characteristics of the upper limbs and their relationship with HGS in volleyball players. However, most research has been conducted in general athlete populations (i.e., inter-university, elite, and female volleyball players) without considering differences across playing positions or providing clear links to functional performance metrics [13,14,15]. Consequently, these findings may not be directly applicable to U-23 national-level athletes, especially in the Colombian context, where talent selection processes may differ.

While upper limb strength is crucial for ball handling and attack efficiency, lower limb performance and static balance are equally essential for maintaining postural control and executing explosive movements [16,17,18,19,20,21]. Effective postural control in static positions and optimal plantar pressure distribution has been associated with enhanced neuromuscular efficiency, improved movement precision, and greater resilience to injury [22,23]. In volleyball athletes, deficits in static balance have been linked to an increased risk of lower extremity injuries (i.e., ankle sprains and functional ankle instability) [24] due to the sport’s high demands for precise body positioning, dynamic control of the center of gravity, and a high degree of ankle stability [19,20].

In this sense, baropodometric analysis provides a detailed understanding of static postural control and plantar distribution by mapping pressure across the plantar surface, which reflects key postural variables [21,25,26]. This computerized system captures plantar imprints and ground reaction forces during quiet standing, dividing the measurements between the right and left feet and further subdividing them into the forefoot, midfoot, and rearfoot [27]. Moreover, it classifies foot types and provides stabilometric parameters based on the spatial and temporal behavior of the center of pressure [28]. These insights allow sports professionals to identify athletes at higher risk of injury early and support the development of targeted proprioceptive and neuromuscular training programs aimed at risk reduction [23,29,30]. Furthermore, monitoring static balance throughout the competitive season yields valuable data for fatigue management and rehabilitation progress, highlighting its relevance for both performance and promoting athlete health.

Despite its proven value in clinical and rehabilitation settings, the application of static baropodometric analysis in volleyball remains limited, and there is still a lack of sport-specific evidence exploring baropodometric characteristics across playing positions [31,32]. This aspect is particularly pivotal in a team sport like volleyball, where positional differences impose distinct physical demands due to the varying requirements of game phases, technical and tactical strategies, and risk of injury associated with each playing position [19,20].

Furthermore, research exploring these variables remains scarce among Latin American volleyball players, especially in the Colombian U-23 male population. This category represents a transitional phase toward elite competition, during which players consolidate technical and tactical skills and physical capacities [2,33]. In Colombia, this category represents a critical talent pool for national and professional teams yet remains understudied. Addressing this gap is essential for developing position-specific profiling to identify talent in the region, assess strengths and weaknesses based on playing position, and implement targeted training programs aimed at improving performance and reducing injury risk [34,35,36].

Given these considerations, the first purpose of the present study was to investigate the anthropometric, baropodometric, and HGS characteristics of male Colombian volleyball players, considering potential differences based on playing position. The second purpose was to assess the influence of upper limbs anthropometric parameters on HGS to better understand the relationship between segmental dimensions and functional performance in volleyball athletes.

## 2. Materials and Methods

### 2.1. Subjects

The present study employed a cross-sectional analytical design and was conducted during the qualification phase of the U-23 Men’s National Volleyball Championship, held in July 2022 in Bucaramanga, Colombia. All eligible athletes participating in the competition were included in the study.

A total of ninety-two male volleyball players from the regional teams of Santander (*n* = 13), Antioquia (*n* = 14), Valle (*n* = 13), Caldas (*n* = 14), Nariño (*n* = 12), Atlántico (*n* = 14), and Cesar (*n* = 12) participated in the study (age: 20.39 (1.74) yrs; height: 184 (8.46) cm; weight: 75.52 (10.20) kg). Players were categorized according to their playing position as setters (*n* = 15), outside (*n* = 21), opposites (*n* = 22), middle blockers (*n* = 19), and liberos (*n* = 15).

All participants were healthy and free from any neuromuscular, orthopedic, or neurological conditions that might interfere with their sports performance, hand function, anthropometric characteristics, or activities of daily living. All participants were thoroughly informed about the study, including the risks and benefits of participation, and if, after this explanation, their decision was not to be included in the analysis, this did not adversely affect any current or future team selection. All included athletes provided written informed consent for testing and data. This study was conducted following the ethical principles outlined in the Declaration of Helsinki. The Ethics Committee for Human Subjects of the University approved this study research (0010-2022/2 May 2022)

### 2.2. Testing Procedures

Comprehensive details regarding the anthropometric parameters, baropodometric evaluations, and HGS tests are provided in the subsequent section. All assessments were performed before training sessions in a private setting at the Bicentenario Volleyball Coliseum during morning hours (between 8:00 and 11:00 a.m.).

The evaluations were carried out in an environment without climate control and followed a predefined order: body composition, upper limb anthropometric measurements, baropodometry, and finally, the HGS assessment. The dominance of the upper and lower limbs was determined by asking the athletes which arm/leg they would use to throw/kick a ball [37,38,39].

Assessments were conducted by two researchers, each with nine years of experience in sports research. Anthropometric measurements were collected by a level 2 anthropometrist. The intraclass correlation coefficient (ICC) values for intra-rater reliability ranged from 0.91 to 0.96, indicating excellent measurement consistency. HGS assessments were conducted by the other researcher, who underwent training that included protocols for the participant’s position and verbal encouragement. The intra-rater reliability showed ICC values of 0.98 for the dominant hand and 0.97 for the non-dominant hand, exhibiting very high reliability (ICC > 0.90) [40].

### 2.3. Body Composition and Anthropometric Measurements

A certified level 2 anthropometrist conducted the measurements following the standardized protocols established by the International Society for the Advancement of Kinanthropometry (ISAK). The mean of two repeated measurements was calculated for each anthropometric variable for data analysis. Using a mechanical stadiometer platform (Seca^®^ 274, Hamburg, Germany; Technical Error of Measurement = 0.019%), height measurements were obtained with participants standing barefoot. The adjustable headpiece was carefully lowered to contact the vertex of the head while the participant performed a deep inhalation. All measurements were taken in meters and rounded to the nearest 0.5 cm.

A bioelectrical impedance analysis (BIA) device (TANITA BC 240 MA, Arlington Heights, IL, USA) was used to assess body composition, with results rounded to the nearest 0.1 unit. Prior to the assessment, participants were instructed to remove any metal objects, abstain from caffeine or diuretics for at least three hours, and void their bladder within 30 min before the testing. The variables collected included weight and body fat percentage (BF%). The body mass index (BMI) was computed as weight (kg)/stature (m^2^).

### 2.4. Upper Limbs Anthropometric Measurements

Participants wore minimal clothing and remained barefoot during the anthropometric measurements to ensure accuracy. A segmometer, a steel tape, a small bone anthropometer, and a skinfold caliper (Cescorf, Porto Alegre, Brazil) were employed to evaluate upper limb lengths, circumferences, and diameters and skinfold triceps thickness, respectively. All upper limb measurements were recorded to the nearest 0.1 cm. For each upper limb, the following parameters were assessed: arm and forearm length, hand breadth, hand length, first-to-fifth finger distance, as well as arm circumference, elbow, and wrist diameters according to the International Anthropometric Standardization Manual edited by ISAK [41].

**Figure 1:**
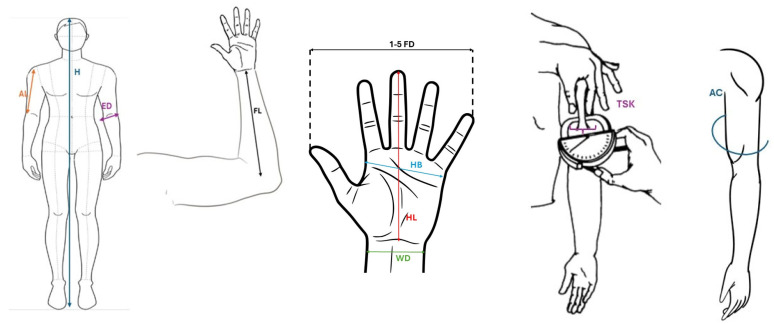
(AL) Arm length. (H) Height. (ED) Elbow length. (FL) Forearm length. (WD) Wrist diameter. (HL) Hand length. (HB) Hand breadth. First–to–fifth finger distance (1–5 FD). Triceps skinfold (TSK). Arm circumference (AC).

Additionally, the triceps skinfold was measured following the standard technique [42], as illustrated in Figure 1.

*Arm length* was assessed as the straight-line distance between the marked acromial point and the radiale, with the participant standing upright, arms relaxed at the sides, and palms resting against their thighs. *Forearm length* was determined by measuring the distance from the radiale to the stylion, with the elbow flexed and the tape positioned parallel to the longitudinal axis of the radius.

*Hand length* was assessed as the shortest distance from the marked mid-stylion line to the dactylion, while *hand breadth* was measured as the span between the radial side of the second metacarpal joint and the ulnar side of the fifth metacarpal joint [43]. The *first-to-fifth finger distance* was recorded as the linear measurement from the outer border of the tip of the thumb to the outer border of the little finger. The fingers and thumb are stretched as widely apart as the person finds comfortable [4].

*Arm circumference* was determined by identifying the midpoint of the distance between the acromial process and the radiale, ensuring proper alignment with the medial and lateral borders of the humerus. The participant stood in a relaxed, upright position with arms hanging naturally by their sides. In the same posture, the *triceps skinfold thickness* was measured by identifying the midpoint between the acromial and the olecranon processes. The skinfold caliper was positioned perpendicular to the fold, approximately 1 cm below the fingers holding the skin. The measurement was recorded 2 s after applying the caliper’s pressure. If two consecutive measurements differed by more than 0.2 mm, a third measurement was taken, and the two closest values were averaged to enhance reliability.

*Elbow diameter* was assessed by measuring the distance between the medial and lateral epicondyles of the humerus. *Wrist diameter* was determined as the linear distance between the outer edges of the radial and ulnar styloid processes.

The arm muscle circumference, arm area, and arm muscle area were calculated according to the formulas outlined in Table 1.

### 2.5. Baropodometry Assessment

Baropodometric measurements were acquired using the electronic portable pressure platform Ecowalk (Ecosanit, Ecotechnology, Inc., Anghiari, Italy). The platform operates at a sampling frequency of 100 Hz and incorporates approximately 1–2 sensors per square centimeter, ensuring high spatial resolution during data acquisition. Data were processed using EcoFoot 4.0 software. Before measurement, participants stood quietly on the platform for approximately 60 s to perform the calibration.

**Figure 2:**
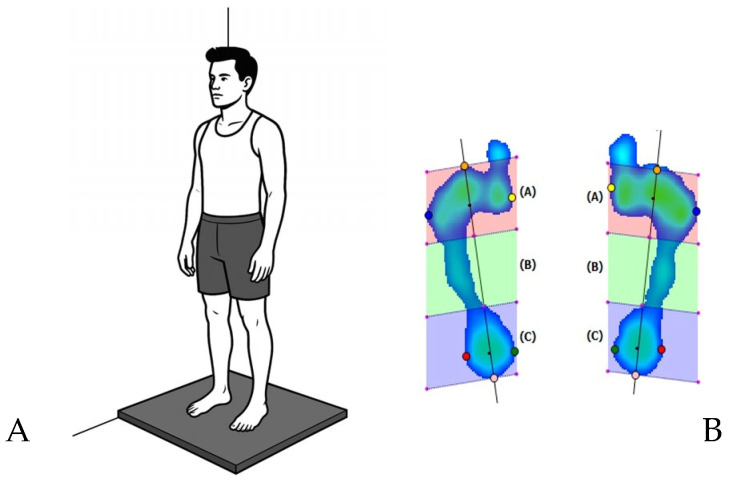
(**A**) Participant position during baropodometry assessment. (**B**) Static analysis of plantar pressure maps using EcoFoot 4.0 software. (A), (B), and (C) zones in (**B**) refer to forefoot, midfoot, and rearfoot zones.

During the measurement process, participants maintained a static bipedal stance on the platform for 20 s, maintaining a forward gaze and barefoot posture (Figure 2A).

Their feet were positioned side by side, and their arms were relaxed along the trunk. Gaze was fixed on a visual marker placed at eye level on the wall, approximately two meters away, to ensure standardized head and neck positioning. This controlled posture was employed to minimize measurement variability. Each athlete underwent two consecutive trials, separated by a one-minute rest interval. The mean value of the two trials was used for subsequent analysis [46].

The following parameters were evaluated for both feet: 95% confident ellipse area, percentage of load distribution, surface area, peak pressure, plantar arch index, and calcaneus angle (Figure 2B).

### 2.6. Handgrip Strength Assessment Protocol

Maximal HGS was measured on both hands using a portable digital hand dynamometer (Takei 5401; Tokyo, Japan) with a precision of 0.1 kg. Participants stood upright during the assessment, with the test arm’s shoulder adducted and the elbow flexed at a 90° angle. The forearm and wrist were maintained in a neutral position to maintain proper alignment of the hand with the forearm during grip assessment. The dynamometer was tailored to each participant’s hand size to promote proper flexion of the metacarpophalangeal joints. Before testing, participants were provided with standardized verbal instructions and verbal encouragement was given throughout the procedure to guarantee maximal effort [2].

A total of three maximum voluntary contractions were performed per hand, with each trial lasting between 3 and 5 s. A 60 s rest interval was allowed between attempts to reduce the risk of fatigue. HGS values were expressed in kilograms (kg), and the highest value for the three trials for each hand was used for statistical analysis [47,48].

### 2.7. Statistical Analysis

All statistical analyses were conducted using the Statistical Package for Social Sciences (SPSS) software v.25 for Mac OS (IBM, Armonk, NY, USA). The assumptions of normality and homoscedasticity were assessed using the Shapiro–Wilk and Levene tests, respectively.

T-paired test, analysis of variance (ANOVA) with a Bonferroni post hoc test, or Kruskal–Wallis with multiple pairwise comparisons was used to determine the differences in anthropometric, baropodometric, and HGS measures between playing positions in volleyball. All data are presented as mean and standard deviation.

Pearson’s and Spearman’s correlation coefficients were used to analyze the relationship between dominant and non-dominant HGS and their corresponding side upper limb anthropometric variables. Based on the conventional approach to interpreting a correlation coefficient [49], coefficients are categorized as “negligible” (r = 0.00–0.10), “weak” (r = 0.10–0.39), “moderate” (r = 0.40–0.69), “strong” (r = 0.70–0.89), and “very strong” (0.90–1.00). Additionally, r-scores were used to identify multicollinearity and shared variance between the variables.

Effect sizes (ES) were reported to measure the magnitude of observed differences. For comparisons involving three or more independent groups, Epsilon Squared (ε^2^) (derived from the Kruskal–Wallis test) and Eta Squared (η^2^) (derived from the ANOVA test) were used to indicate the proportion of variance explained. Interpretation of ES was based on conventional thresholds: Epsilon Squared: small (ε^2^ < 0.01), moderate (ε^2^ = 0.01–0.06), and large (ε^2^ ≥ 0.06). Eta Squared: small (η^2^ = 0.01), medium (η^2^ = 0.06), and large (η^2^ = 0.14) [50].

A stepwise multiple linear regression analysis was performed to identify the most relevant upper limb anthropometric variables associated with dominant HGS. To reduce the risk of overfitting given the sample size [51], the number of covariates was restricted to a maximum of nine, selecting those with the strongest correlation coefficients with HGS. The stepwise method followed entry and removal criteria based on significance levels (*p* < 0.05 and *p* > 0.10, respectively). Statistical significance was set at *p* < 0.05.

## 3. Results

Descriptive statistics for the sample, including anthropometric characteristics and HGS of both upper limbs in U-23 male volleyball players by playing position, are summarized in Table 2. One-way ANOVA revealed significant differences for player position for body height (*F*_(4, 87)_ = 6.13; *p* < 0.001) and body fat percentage (*F*_(4, 74)_ = 2.76; *p* < 0.05), with outsides, opposites, and middle blockers exhibiting the largest height and lower body fat percentage than liberos.

Similarly, the analysis also showed significant differences on the dominant side for several variables. For HGS (*F*_(4, 87)_ = 2.93; *p* < 0.05), opposites outperformed setters. For arm length (*F*_(4, 87)_ = 4.53; *p* < 0.01) and forearm length (*χ*^2^_(4)_ = 22.65; *p* < 0.01), middle blockers outdid setters and liberos in both variables. Significant differences were also found for wrist diameter (*χ*^2^_(4)_ = 18.59; *p* < 0.01) and hand length (*χ*^2^_(4)_ = 18.02; *p* < 0.01), where opposites and middle blockers outperformed liberos. Regarding hand breadth (*F*_(4, 87)_ = 3.66; *p* < 0.01), outsides, middle blockers, and opposites exhibited greater values than those of the liberos. Finally, for the 1–5 finger distance (*χ*^2^_(4)_ = 11.40; *p* < 0.05), middle blockers outperformed liberos.

On the non-dominant side, significant differences were found for several anthropometric variables. Arm length (*F*_(4,87)_ = 5.08; *p* < 0.01) and wrist diameter (*χ*^2^_(4)_ = 20.06; *p* < 0.01) were greater in opposites, outsides, and middle blockers compared to liberos. Differences were also observed in forearm length (*χ*^2^_(4)_ = 24.54; *p* < 0.01), with opposites, outsides, and middle blockers outperforming both liberos and setters. In hand length (*χ*^2^_(4)_ = 12.94; *p* < 0.05) and hand breadth (*F*_(4, 87)_ = 2.72; *p* < 0.05), middle blockers and opposites outperformed liberos, respectively. Lastly, the 1–5 finger distance was significantly larger (*χ*^2^_(4)_ = 17.67; *p* < 0.01) in opposites, outsides, and middle blockers than in liberos.

**Figure 3:**
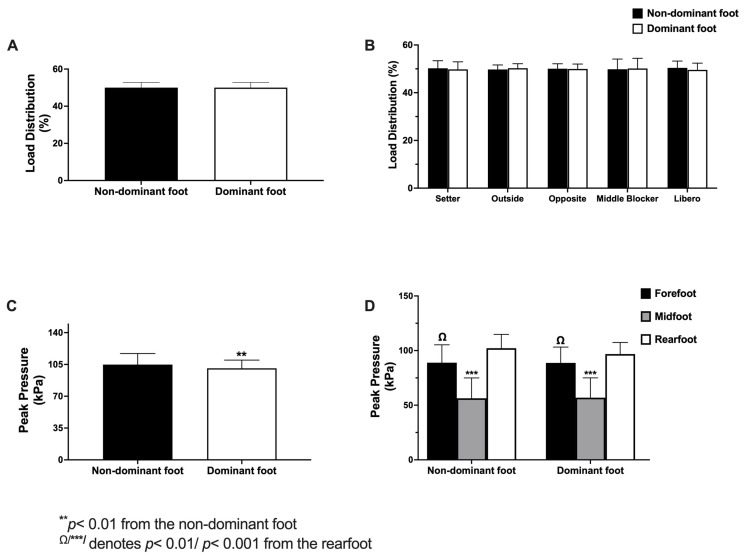
Analysis of load percentage and peak pressure distribution. (**A**) Distribution of load percentage between the feet. (**B**) Load percentage distribution by position. (**C**) Distribution of peak pressure between the feet. (**D**) Distribution of peak pressure according to foot zones. Statistical significance was assessed using a T-paired test and one-way ANOVA for (**A**), (**C**), and (**B**), (**D**), respectively.

Analysis of load percentage distribution, which refers to which foot each player carries the most weight, showed no differences between feet (*t*_(65)_ = 1.07, *p* > 0.05) (Figure 3A) or between positions in the non-dominant foot (*F*_(4, 61)_ = 0.115; *p* > 0.05) or the dominant foot (*F*_(4, 61)_ = 0.113; *p* > 0.05) (Figure 3B). After that, we analyzed the behavior of peak pressure on the feet. T-paired test revealed higher levels on the non-dominant foot (*t*_(65)_ = 2.67, *p* < 0.01) (Figure 3C). Then, when we analyzed peak pressure levels according to the zone on foot, which revealed greater levels in the forefoot and rearfoot zones of the non-dominant (*F*_(2, 195)_ = 142.17; *p* < 0.01) and dominant (*F*_(2, 195)_ = 135.20; *p* < 0.01) feet than in the midfoot zone. Likewise, the rearfoot zone exhibited higher pressure levels than those found in the forefoot zone (*p* < 0.01) in both feet, as shown in Figure 3D. Finally, we also analyzed the peak pressure levels according to the position of players, with no differences detected in either foot.

Additionally, the analysis of other baropodometric parameters, including the ellipse area, calcaneus angle, and total foot surface, showed no statistically significant differences in the whole sample, even when the between-group position factor was considered (Table S1).

Table 3 presents the correlations between selected upper limb anthropometric variables measured in the study with dominant and non-dominant HGS in U-23 male volleyball players. We found moderate and weak positive correlations between most anthropometric variables and HGS on both sides. Furthermore, no significant correlations were identified with the hand shape index for the dominant (r_(91)_ = 0.09; *p* > 0.05) and non-dominant side (r_(91)_ = −0.02; *p* > 0.05).

Finally, a multivariate analysis was conducted to evaluate whether selected anthropometric parameters could explain dominant HGS (Table 4). Based on the stepwise multiple linear regression applied to the nine variables with the strongest correlation with dominant HGS (Table 3), three variables were retained in the final model. Specifically, the analysis showed that dominant hand breadth (β = 3.42, 95% CI [0.43, 6.40]), dominant upper arm muscle area (β = 0.157, 95% CI [0.02, 0.29]), and dominant wrist diameter (β = 3.59, 95% CI [0.49, 6.68]) entered the regression equation and explained 27.5% (R^2^ = 0.30; adjusted R^2^ = 0.27) of the variance in dominant HGS (F_(3,88)_ = 12.53; *p* < 0.001).

## 4. Discussion

This cross-sectional study provides a comprehensive analysis of the morphological characteristics, baropodometric variables, and HGS performance of U-23 male Colombian volleyball players. Notably, significant differences (*p* < 0.05) were observed in all measurements of lengths and breadths of both the dominant and non-dominant upper limbs among players, according to their position. Furthermore, an association was found between all hand, forearm, and arm dimensions with HGS on both sides, identifying selected upper limb dimensions as moderately associated variables of HGS. Significant differences (*p* < 0.05) were also found in some baropodometric measures between feet and across different foot regions in both feet.

Anthropometric characteristics have been extensively investigated in volleyball, with most studies focusing on describing players’ somatotypes and body fat percentage. However, specific upper limb dimensions, such as the lengths, diameters, and breadth of arms, forearms, and hands, have received less attention [2].

Research has shown position-specific descriptions of anthropometric and physical performance in volleyball players [19,52]. Thus, middle blockers have been described as the tallest, most ectomorphic, least mesomorphic, and endomorphic. At the same time, liberos are more likely to be shorter, less ectomorphic, more mesomorphic, and endomorphic than players in other positions [53]. In this sport, certain anthropometric and morphological characteristics (e.g., height, weight, body composition, arm, forearm, and hand dimensions) are essential for success during the game. For instance, serves, blocks, and spikes are performed more efficiently when players possess larger upper limb dimensions [53].

Results of the present study showed that significant differences exist among volleyball players of different playing positions. Therefore, opposites, middle blockers, and outsides were taller, had a lower body fat percentage than liberos, and exhibited higher values in most of the selected upper limb variables (upper arm length, forearm length, wrist diameter, hand length, and hand breadth) than the liberos and setters (Table 2). Similar results to those reported in our study have been found by other researchers [19,52,54]. For instance, the latest findings of Milić et al. (2024) [19] on female volleyball players described liberos and setters as the smaller players in the team. These findings were also reported by Toselli et al. (2018) [52] in a sample of elite male volleyball players. Researchers also observed great differences in upper limb dimensions (arm’s length and humerus width) among the players of the different roles, with opposites and middle blockers outdoing setters and liberos, respectively.

Although height is a critical factor in volleyball success, as it enhances a player’s ability to compete effectively over the net, liberos are not required to be taller. Moreover, height does not seem critical for them since they play in the back row and are not allowed to spike or block. Instead, having a low center of mass is critical for effectively handling low balls during landings, receiving, and defense, which suits shorter players [13]. Thus, the shorter height of libero players in this study is aligned with these roles in volleyball. Conversely, other positions (opposites, middle blockers, and outsides) require taller players with larger upper limb lengths to be successful during spiking and blocking [55]. These anthropometric characteristics enable players to extend their reach, allowing them to contact the ball over the top of the opponent’s block and from a higher point, thereby increasing the ball’s downward trajectory and speed [56].

While a greater height provides better reach above the net, facilitating easier control of defensive and offensive actions, a broader wrist allows for more forceful hits during attacks. Additionally, longer arms have been related to better performance over the net in both attacking and defensive actions [55].

Liberos and setters are key volleyball positions that require agility, precision, and technical skills rather than power or extended reach. Liberos are defensive specialists whose primary responsibilities demand quick lateral movements and a low body position to respond to the ball effectively. On the contrary, extending upward or outward for blocks or spikes is not a function of this position [57,58]. To succeed in most of the actions, liberos must often maintain a low center of gravity, which could be supported, in part, by having shorter arm spans and smaller upper limb dimensions [19,59].

Setters, meanwhile, focus on ball distribution and coordination, requiring fast, accurate hand movements rather than the extended reach needed for offensive or defensive net play. Because decision-making is the main ability expected from athletes in this position [60], the setter plays an essential role in a volleyball team to effectively organize offensive strategies [61,62]. In this regard, having smaller upper limb dimensions could facilitate rapid arm motions, which are necessary to set the ball accurately under time constraints. Along with this, players in this position rarely spike during a match [63,64], so high levels of upper body power may also not be necessary.

Here, setters also showed the lowest values in the dominant HGS among the different positions, being statistically outperformed by the opposites (Table 2). As previously mentioned, players in this position do not rely heavily on upper limb strength as their roles emphasize ball control, agility, and decision-making. For that reason, it would be reasonable for these players to have lower HGS values compared to those in offensive positions. Despite several studies focusing on strength in male volleyball players, there is little published research evaluating upper limb strength. Consistent with the findings of our study, Toselli et al. (2018) [52] also observed lower HGS scores in setters compared to opposite players. Similarly, the González-Badillo group also described worse strength performance in setters compared with opposites and middle blockers in a 4RM bench press test [65].

Our findings can be partially explained by the geometric scaling paradigm [65,66], which has been successfully used to examine the influence of body size on athletic performance. According to this paradigm, strength is closely linked to the muscle cross-sectional area, which increases with increases in body height. This suggests that individuals with greater limb dimensions would be likely to excel in activities that require a strength component [65]. Thus, this theory is supported in the current study by the significant positive correlations observed between height and selected upper limb dimensions with the dominant HGS (Table 3).

In addition, hand breadth, upper arm muscle area, and wrist diameter were identified as anthropometric variables that explain 27% of the variance of HGS (Table 4). These findings are consistent with the existing literature, which underscores the critical role of larger hand dimensions and upper arm musculature in determining HGS, a key factor influencing various technical movements in volleyball [53,67]. Nevertheless, this association should be interpreted with caution due to the explained variance being modest, indicating that other factors, such as neuromuscular control or sport-specific strength, likely play an important role.

Fallahi et al. (2011) [53] suggest that larger hand anthropometric variables lead to a reduced finger spread, thereby enhancing grasp efficiency and reducing fatigue during ball manipulation. Additionally, wrist diameter, which reflects bone structure and muscular support, may contribute to greater force application in repeated gripping actions. This biomechanical advantage allows players to exert better control over the ball during overhead passes and serves, improving precision and consistency [2,68]. Furthermore, the upper arm muscle area serves as an indirect marker of overall upper limb strength. This characteristic is essential for executing blocks and spikes with maximal power while also enabling athletes to withstand high-impact forces [69].

Building upon this evidence, these parameters could be valuable for identifying talent and guiding coaching strategies in volleyball. Integrating HGS-related parameters into scouting and training processes may provide a more objective approach to assessing players’ physical potential and skill development.

Regarding the baropodometric profile results, no significant differences were observed in the percentage of load distribution between the feet or across playing positions (Figure 3A,B). Similarly, when analyzing balance-related variables such as the ellipse area, no positional differences were found (Table S1), which aligns with the results of load percentage distribution. This finding may be attributed to the fact that when load distribution is balanced, oscillations in the center of pressure (CoP) are minimized, resulting in a reduced ellipse area, which reflects greater postural efficiency [25,70]. In our study, the sample consisted of high-level players in each position, suggesting a relatively homogeneous postural control and stability.

Interestingly, when the entire sample was analyzed, significant differences in peak pressure were observed between feet, with the non-dominant foot experiencing higher pressure levels (Figure 3C). However, no significant differences were found when peak pressure was analyzed across playing positions. One possible explanation for this asymmetry could be variation in the plantar surface area among players, as pressure is inversely proportional to this variable [71]. Nevertheless, our analysis revealed no significant differences in plantar surface area between feet (Table S1), suggesting that other biomechanical or neuromuscular factors may contribute to this discrepancy, as has been suggested in previous biomechanical studies [72,73]. However, this hypothesis requires confirmation through dynamic or kinematic assessments.

As reported in the literature [71,74], volleyball-specific movements may contribute to plantar pressure asymmetry. The frequent jumps, lateral movements, and unbalanced landings could explain the higher pressure observed in the non-dominant foot, given its stabilizing role, particularly when setting up for a spike or block. This repeated exposure to sport-specific loading patterns may reinforce the asymmetrical pressure distribution during movements, as previously reported by our research group [46,75]. Moreover, our findings regarding pressure distribution across foot regions (Figure 3D) align with previous evidence indicating that, in a normal foot, pressure is typically distributed as 60% in the rearfoot, 30% in the forefoot, and 10% in the midfoot [76,77]. This distribution is supported by the fact that 80% of our sample exhibited this kind of foot, reinforcing the consistency between expected biomechanical pressure patterns and the observed data.

Finally, the identification of upper limb-associated anthropometric variables of HGS and the baropodometric profile across playing positions could be integrated into an athlete’s screening protocol and talent scouting, serving as practical applications of these findings. Additionally, these results may contribute to the development of injury prevention strategies by identifying potential asymmetries in plantar pressure distribution. Such insights may be crucial for managing overuse injuries and biomechanical inefficiencies.

This study provides a comprehensive assessment of anthropometric, baropodometric, and HGS characteristics across playing positions in a homogeneous sample of high-level U-23 Colombian male volleyball players. Consequently, this research offers a broad and in-depth perspective on the physical attributes that contribute to volleyball success.

However, these results may not be directly applicable to younger players, female athletes, or recreational volleyball players, as the participants in the present study are highly trained individuals. The sample size of the study was influenced by the availability of participants in the competition, which may have limited the detection of subtle effects and affected the generalizability of our findings. Furthermore, the exclusive use of static baropodometric assessment limits the generalizability of postural control findings to dynamic, sport-specific contexts. Additionally, the modest associations observed between anthropometric variables and HGS should be interpreted with caution, as they indicate that other physiological or biomechanical factors likely contribute to strength performance. Future research should employ longitudinal studies to explore how these variables evolve with training and their impact on injury risk and performance over time.

## 5. Conclusions

This study provides the first characterization of anthropometric, HGS, and baropodometric parameters in U-23 Colombian male volleyball players, highlighting position-specific differences. Positional specific differences were observed in upper limb morphology, with opposites, outsides, and middle blockers showing greater segmental dimensions and HGS compared to setters and liberos. Selected upper limb parameters such as hand breadth, upper arm muscle area, and wrist diameter were moderately associated with HGS. Although baropodometric parameters did not differ by playing position, foot dominance and specific zones were linked to changes in load distribution and peak pressure.

These findings suggest that positional profiling in volleyball provides critical insights into the physical attributes necessary for optimizing performance and injury prevention. The identification of moderately associated anthropometric variables with HGS, along with baropodometric pressure patterns, might contribute to the development of position-specific training and screening strategies.

## Figures and Tables

**Table 1:** Formulas to estimate upper arm anthropometric variables.

Anthropometric Variables	Formula
Arm muscle circumference (cm)	S = c − (T × 3.14) [44]
Arm area (cm^2^)	A = c^2^/12.56 [45]
Arm muscle area (cm^2^)	M = S^2^/12.56 [44]

S: arm muscle circumference; c: arm circumference; T = triceps skinfold; A: arm area; M: arm muscle area.

**Table 2:** Descriptive statistics of anthropometric and HGS variables in U-23 Colombian male volleyball players (*n* = 92).

Variable	Setter (*n* = 15) ^1^	Outside (*n* = 21) ^2^	Opposite (*n* = 22) ^3^	Middle Blocker (*n* = 19) ^4^	Libero (*n* = 15) ^5^	Effect Size
Body weight (Kg)	74.03 (11.38)	76.42 (8.07)	76.84 (8.04)	77.34 (11.57)	73.66 (12.40)	0.22
Height (cm)	181.10 (10.28) **^4^**	183.82 (5.75)	185.30 (7.86)	189.43 (7.58)	174.48 (6.84) **^2,3,4^**	
Body fat (%)	8.27 (3.82)	10.29 (3.87)	7.95 (2.62)	7.66 (3.80)	11.28 (4.80)	0.13
Body water (%)	63.58 (2.96)	62.12 (2.61)	63.54 (2.48)	63.95 (5.00)	61.68 (2.84)	
**Upper limbs anthropometric variables—Dominant side**						
Upper arm length (cm)	34.58 (2.81)	35.89 (2.05)	35.51 (2.12)	36.81 (2.19) **^1,3^**	33.16 (2.04)	0.17
Arm circumference (cm)	29.58 (2.48)	30.14 (2.54)	29.58 (2.19)	29.54 (2.80)	30.42 (3.04)	
Forearm length (cm) ^†^	25.66 (1.30)	27.11 (1.28)	26.68 (2.88)	28.37 (1.42) **^1,3^**	25.58 (1.24)	0.20
Elbow diameter (cm)	6.86 (0.40)	6.94 (0.43)	6.98 (0.35)	7.08 (0.29)	6.75 (0.26)	
Wrist diameter (cm) ^†^	5.65 (0.41)	5.73 (0.27)	6.01 (0.77) **^5^**	5.85 (0.36) **^5^**	5.51 (0.19)	0.16
Hand length (cm) ^†^	19.70 (1.13)	19.22 (4.30)	20.13 (1.07) **^5^**	20.93 (1.19) **^5^**	19.00 (0.64)	0.15
Hand breadth (cm)	8.34 (0.50)	8.59 (0.48) **^5^**	8.50 (0.56) **^5^**	8.73 (0.35) **^5^**	8.20 (0.32)	0.14
1–5 finger distance (cm) ^†^	21.79 (1.42)	22.50 (1.49)	22.19 (1.21)	22.69 (1.81) **^5^**	21.08 (1.14)	0.08
Upper arm muscle area (cm^2^)	58.01 (11.39)	59.37 (9.51)	59.93 (10.05)	58.99 (10.09)	61.10 (9.78)	
Upper arm muscle circumference (cm) ^†^	26.88 (2.48)	27.2 (2.17)	27.3 (2.32)	27.1 (23.10)	27.6 (21.74)	
Upper arm area (cm^2^)	70.12 (12.05)	72.80 (12.09)	70.02 (10.21)	70.07 (13.24)	74.37 (15.29)	
Handgrip strength (kgf)	41.39 (6.82) **^3^**	44.87 (7.32)	48.65 (8.58)	47.10 (5.05)	43.81 (4.86)	0.12
**Upper limbs anthropometric variables—Non-dominant side**						
Upper arm length (cm)	34.33 (2.80)	35.58 (1.63)	35.60 (1.93)	36.34 (2.20)	32.96 (2.13) **^1–4^**	0.19
Arm circumference (cm)	29.46 (2.51)	30.07 (2.58)	29.44 (2.25)	28.90 (2.90)	30.00 (3.00)	
Forearm length (cm) ^†^	25.54 (1.86) **^2–4^**	26.77 (1.46)	28.26 (4.05)	28.12 (1.76)	25.08 (1.58) **^2–4^**	0.23
Elbow diameter (cm)	6.75 (0.64)	6.95 (0.47)	7.09 (0.26)	7.11 (2.78)	6.77 (0.28)	
Wrist diameter (cm) ^†^	5.71 (0.84)	5.69 (0.27)	5.73 (0.34)	5.73 (0.39)	5.33 (0.14) **^1–4^**	0.18
Hand length (cm) ^†^	19.58 (1.01)	20.39 (1.05)	20.47 (1.54) **^5^**	20.97 (1.21)	19.25 (1.38)	0.09
Hand breadth (cm)	8.30 (0.41)	8.52 (0.50)	8.50 (0.44) **^5^**	8.63 (0.33)	8.05 (0.30)	0.11
1–5 finger distance (cm) ^†^	21.91 (1.37)	23.25 (1.94)	21.93 (5.22)	23.03 (1.84)	20.91 (1.25) **^2–4^**	0.15
Upper arm muscle area (cm^2^)	57.51 (11.56)	59.23 (9.48)	59.61 (10.29)	55.99 (9.78)	58.75 (9.88)	
Upper arm muscle circumference (cm)	26.88 (2.48)	27.19 (2.17)	27.26 (2.48)	26.42 (2.29)	27.07 (2.27)	
Upper arm area (cm^2^)	69.55 (12.17)	72.50 (12.25)	69.40 (10.68)	67.12 (13.42)	72.35 (14.69)	
Handgrip strength (kgf)	43.03 (8.17)	45.52 (6.64)	49.42 (8.70)	48.61 (6.14)	44.13 (4.46)	

Significantly different from the following: ^1^—setter; ^2^—outside; ^3^—opposite; ^4^—middle blocker; ^5^—libero; ^†^ differences were assessed with Kruskal–Wallis test. All data are presented as mean (standard deviation).

**Table 3:** Pearson’s and Spearman correlation coefficients between all anthropometric variables and HGS in U-23 male volleyball players.

Variables	Dominant HGS	Non-Dominant HGS
Height (cm)	0.33 **	0.43 ***
Upper arm length (cm)	0.30 **	0.47 ***
Arm circumference (cm)	0.33 **	0.41 ***
Forearm length (cm)	0.37 ***	0.43 ***
Elbow diameter (cm)	0.24 *^,†^	0.37 ***
Wrist diameter (cm)	0.42 ***	0.42 ***
Hand length (cm)	0.44 ***	0.47 ***
Hand breadth (cm)	0.47 ***^,†^	0.60 ***
1–5 finger distance (cm)	0.29 **^,†^	0.36 ***
Upper arm muscle area (cm)	0.40 ***	0.49 ***
Upper arm muscle circumference (cm)	0.40 ***	0.48 ***
Upper arm area (cm^2^)	0.33 **	0.42 ***

*/**/***/ denotes *p* < 0.05/*p* < 0.01/*p* < 0.001; **^†^** denotes Spearman correlations. All the anthropometric variables correspond to the same side as the HGS measurement (dominant or non-dominant).

**Table 4:** Stepwise multiple regression analysis between dominant HGS-associated variables in U-23 volleyball players.

Independent Variables	β	t	*p*	95% CI	VIF
Dominant hand breadth (cm)	3.42	2.27	0.025	[0.43, 6.40]	1.43
Dominant upper arm muscle area (cm^2^)	0.157	2.32	0.023	[0.02, 0.29]	1.22
Dominant wrist diameter (cm)	3.59	2.30	0.023	[0.49, 6.68]	1.31

VIF: Variance Inflation Factor.

## Data Availability

The datasets used and/or analyzed during the current study are available from the corresponding author upon reasonable request.

## Supplementary Materials

The following supporting information can be downloaded at https://www.mdpi.com/article/10.3390/jfmk10020197/s1, Table S1: Descriptive statistics of baropodometric variables in Colombian male volleyball players.
