# Supplementary material for: Positional Profiling of Anthropometric, Baropodometric, and Grip Strength Traits in Male Volleyball Players: Insights from a National Colombian Study
Source: J Funct Morphol Kinesiol. 2025 May 29;10(2):197. doi: 10.3390/jfmk10020197 (PMC12193723; doi:10.3390/jfmk10020197)
Supplement: Supplementary file 1 [file jfmk-10-00197-s001.zip › jfmk-3543392-supplementary.pdf]

Supplementary material

Positional Profiling of Anthropometric, Baropodometric, and Grip Strength Traits in Male Volleyball Players: insights from a National Colombian Study.

Journal of Functional Morphology and Kinesioloy

Adrián De la Rosa[1,2,3*†], María Alejandra Camacho-Villa [3,4†], Fernando Millan-Domingo[1], Juan Carlos Saavedra[5], Marina Politi Okoshi[6], Luana Urbano Pagan[6]

1. Freshage Research Group, Department of Physiology, Faculty of Medicine, University of Valencia, CIBERFES, Fundación Investigación Hospital Clínico Universitario/INCLIVA, 46010 Valencia, Spain; adrianmdegonz@outlook.com (A.D.l.R); fernando.millan-domingo@uv.es (F.M.-D).
2. Body, Physical Activity and Sport Study Group (GECAFD), Sports Department, Universidad Industrial de Santander, Bucaramanga 680002, Santander, Colombia
3. HARPEER Research Group, Yumbo 760001, Colombia
4. Pain Study Group (GED), Physical Therapy School, Universidad Industrial de Santander, Bucaramanga 680002, Santander, Colombia; alejacvilla@gmail.com (M.A.C.-V).
5. GCED Research group, Bucaramanga 688006, Colombia; Juancs19@hotmail.com (J.C.S).
6. Botucatu Medical School, São Paulo State University, Botucatu 18618-970, Brazil; luana.pagan@unesp.br (L.U.P); marina.okoshi@unesp.br (M.P.O.).

   *Correspondence: adrianmdegonz@outlook.com

   † These authors contributed equally to this work.

Table S1. Descriptive statistics of baropodometric variables U-23 Colombian male volleyball players

Variable	Setter	Outside	Opposite	Middle Blocker	Libero
95% confident elipse area	45.43 (41.47)	28.58 (18.09)	38.67 (22.19)	50.59 (29.26)	26.33 (17.70)
**Dominant Side**					
Foot load distribution (%)	49.76 (3.18)	50.40 (1.90)	49.94 (2.07)	50.19 (4.46)	49.58 (2.81)
Foot Surface (cm)	115.40 (11.45)	117.84 (14.62)	121.77 (14.90)	125.22 (12.20)	108.90 (8.90)
Foot pressure (Kpa)	99.20 (13.12)	103.92 (7.19)	100.44 (8.41)	97.75 (7.96)	100.18 (6.08)
Arch Index	21.63 (5.68)	20.46 (6.72)	22.84 (5.66)	20.95 (7.67)	22.31 (3.21)
Calcaneus angle (°)	10.14 (5.40)	8.93 (4.50)	10.29 (5.94)	10.09 (5.61)	8.08 (4.72)
**Non-Dominant side**					
Foot load distribution (%)	50.24 (3.18)	49.60 (1.90)	50.06 (2.07)	49.80 (4.46)	50.42 (2.83)
Foot Surface (cm)	116.70 (17.18)	117.30 (17.04)	123.77 (15.25)	123.83 (16.71)	110.36 (90.80)
Foot pressure (Kpa)	104.6 (12.60)	108.23 (10.86)	103.33 (15.33)	104.83 (11.44)	102.09 (9.47)
Arch Index	21.62 (4.04)	19.38 (7.00)	23.34 (3.92)	19.22 (4.84)	23.83 (4.38)
Calcaneus angle (°)	8.50 (4.71)	7.39 (3.90)	9.00 (3.89)	9.77 (7.16)	8.13 (3.50)

All data are presented as mean (Standard Deviation).
